# Crystallizing short-read assemblies around seeds

**DOI:** 10.1186/1471-2105-10-S1-S16

**Published:** 2009-01-30

**Authors:** Mohammad Sajjad Hossain, Navid Azimi, Steven Skiena

**Affiliations:** 1 Department of Computer Science, Stony Brook University, Stony Brook, NY 11794-4400 USA

## Abstract

**Background:**

New short-read sequencing technologies produce enormous volumes of 25–30 base paired-end reads. The resulting reads have vastly different characteristics than produced by Sanger sequencing, and require different approaches than the previous generation of sequence assemblers. In this paper, we present a short-read de novo assembler particularly targeted at the new ABI SOLiD sequencing technology.

**Results:**

This paper presents what we believe to be the first de novo sequence assembly results on *real *data from the emerging SOLiD platform, introduced by *Applied Biosystems*. Our assembler SHORTY augments short-paired reads using a trivially small number (5 – 10) of *seeds *of length 300 – 500 bp. These seeds enable us to produce significant assemblies using short-read coverage no more than 100×, which can be obtained in a single run of these high-capacity sequencers. SHORTY exploits two ideas which we believe to be of interest to the short-read assembly community: (1) using single seed reads to crystallize assemblies, and (2) estimating intercontig distances accurately from multiple spanning paired-end reads.

**Conclusion:**

We demonstrate effective assemblies (N50 contig sizes ~40 kb) of three different bacterial species using simulated SOLiD data. Sequencing artifacts limit our performance on real data, however our results on this data are substantially better than those achieved by competing assemblers.

## Background

Several new short-read sequencing technologies are now actively competing in the race towards the $1,000 genome. Each of these technologies produces raw sequence data with particular characteristics and distinct error models. However, it has become clear that three major contenders (Solexa/Illumina, Agencourt/Applied Biosystems, and Helicos BioSciences) aim to produce high volume, 25–30 base paired-end reads.

Short read sequencing has lead to a new surge of interest in the old problem of sequence assembly. These new technologies have only recently started producing data suitable for de novo assembly. Several teams are now building short-read assemblers (see Section "Related Work"), but the protocols optimizing assembly projects (e.g. optimal mixes of short- and long-reads) are still being invented.

In this paper we present our assembler SHORTY, targeted towards paired-end microread sequencing data. SHORTY uses a very small volume of "seeds", perhaps 5–10 reads/contigs per bacterial genome. These seeds can be either virtually constructed using a conventional single-ended read assembler, or purchased with a trivial amount of Sanger sequencing. Still, these seeds enable us to produce significant assemblies with a short-read coverage of 100×, easily obtainable on one run of any of the new machines. Further, our final assemblies prove very accurate even though our reads contain base error rates associated with the machines available today. Certain previous work on microread assembly underestimates the complexity of the problem by simulating assembly on error-free reads.

SHORTY exploits two ideas which we believe of interest to the short-read assembly community:

• *Seed reads for crystallizing assemblies *– Several other assemblers intermix low (say 2×) coverage from Sanger or 454 reads with a higher coverage of short reads to fill up gaps. Instead, we use a single 300 – 500 base computed seed read to grow a neighboring contig of greater or equal to Sanger length. By repeating this process on the new contig, we can walk across the full genome assembling perhaps 90% of the genome into 15–20 kb contigs. Assembling the results of such walks from a trivial number of seeds produces contigs with an N50 size (length such that at least 50% of the assembled genome lies in blocks of the N50 size or greater) of 40 kb on bacterial genomes and 98% coverage in non-trivial contigs.

The *seed *coverage assumed by our protocol is so modest it eliminates the need for a lab to own more than one type of sequencing platform. These trivial number of reads can be generated from short reads by following any simple assembly algorithm or likely even extracted from highly-conserved ribosomal RNA sequences scavenged from databases. Already existing assemblers (e.g. [1,2]) for single-ended short reads can generate these starter seeds.

• *Inter-contig distance estimation from spanning paired-end reads *– Sequencing protocols specify the mean separation distance and variance between the ends of the paired-end reads. Typically, these insert lengths are normally distributed, say with a mean distance of 2700 bases and a standard deviation of 350 bases. Our walking assembly strategy naturally produces two neighboring contigs separated by some insert distance. The substantial number of paired-end reads with one end anchored in each contig provides the possibility of accurately estimating the distance between the contigs. Such estimation enables us to order contigs and fill gaps using shorter overlaps that would be unconvincing in the absence of distance information.

Huson et. al [3] proposes an idea to order contigs from mate-pair reads but it is not scalable for the volume of data we have in a short read assembly project. In this paper, we use such distance estimation efforts in a way that scales for short read assemblers.

We survey related work on short-read assembly below. The primary research issue today is not the head-to-head comparison of which assembler is "best", but to identify the most cost-effective short-read sequencing protocol which results in data that can be reconstructed when coupled with the right assembly strategy.

### Short read sequencing technologies

Although the Sanger sequencing method [4] has been the dominant sequencing technology for decades, novel technologies for short read sequencing are being developed by several groups. [5-9]. See [10] for a recent survey and analysis of these technologies. These new short-read sequencing technologies differ in details of localizing molecules, amplification and sequencing approach. Our assembler has been developed for microread technologies that generate mate paired short reads. Hence, it is suitable for data generated by companies like:

• *Applied Biosystems*: They recently released their SOLiD™ sequencing machine, which uses technology that is acquired from Agencourt Bioscience Corporation. Agencourt commercialized their technology based on *Polony Sequencing *developed by Church and Mitra [11,12]. Indeed, the parameters underlying our simulations were selected with SOLiD™ in mind.

• *Solexa*: They were recently acquired by *Illumina*. Their sequencing machine, *Illumina Genome Analyzer*, can load to eight samples onto their flow cell surface for simultaneous analysis. The platform offers high accuracy, high throughput and relatively low cost ($3000 per run, $400 per channel), and promises real support for double-ended reads forthcoming very soon.

• *Helicos BioSciences*: They are pioneering a single-molecule approach to sequencing based on technology from [13]. This offers advantages in capacity and eliminating amplification-specific bias. Their HeliScope™ sequencing machine contains two flow cells where billions of single molecules of sample DNA are captured on an application-specific proprietary surface to serve as templates for the sequencing-by-synthesis process. Recently, they published [14] M13 genome re-sequencing data based on their new technology.

Table 1 compares the primary performance characteristics of various short read sequencing technologies.

**Table 1:** Comparison of different short read sequencing technologies. Comparison of existing short read sequencing technologies in terms of throughput and read length.

Company	Machine	Throughput (per run)	Read length (base)
454	GS FLX	100 M bases/7 hours	100 or more
Helicos	HeliScope	2 G bases/day	around 25
Applied Biosystems	SOLiD	4 G bases	25 – 30
Solexa	Illumina Genome Analyzer	2 G bases/2 days	25 – 30

### Related work

The success of shotgun sequencing [4] led to the development of several successful assemblers for Sanger reads. Most of them were based on the overlap-layout-consensus [15] paradigm, while others took a graph-theoretic approach. Some assemblers were suitable for hierarchical sequencing, while others targeted *whole genome shotgun *(WGS) sequencing.

As short read sequencing technologies mature, several bioinformatics groups have started working on short read assembly projects. Most algorithms are still tested on simulated data, as true assembly-quality data is not yet readily available for most platforms. Solexa double-ended reads and Applied Biosystems' SOLiD™ system have just entered the market, so real data should be available in relatively short order. We classify short read assemblers in three different groups, based on the type of reads they expect. The first class of assemblers are similar to ours in targeting mate-paired data generated from Solexa/ABI machines:

• ALLPATHS [16] is an assembler being developed at the *Broad Institute *reporting excellent assembly on paired-end Solexa-type data with 80× coverage using a protocol with three different insert sizes (50 kb ± 10%, 6 kb ± 10%, and 0.5 kb ± 1%). SHORTY is different in (1) assuming a substantially simpler, single library experimental protocol and (2) employing shorter reads (25 vs. 30 bp).

• Medvedev and Brudno's RECOMB 2008 paper [17] reports assembly results for bacterial scale genomes which are more directly comparable to ours. They assemble simulated 25-base paired (although error-free) reads into contigs with N50 contig sizes around 25 kb. We produced bigger N50 (30 – 45 kb) even with the presence of sequencing errors.

• *Velvet *[18] augments 50× Solexa data to produce high quality assemblies. They use reads of 35 bp length whereas SHORTY can handle 25 bp long reads, which is more realistic for SOLiD data. Our N50 size is comparable with those of *Velvet *despite the shorter read length.

The second class are assemblers targeting 454 data, which include:

• *Newbler *[19] is a proprietary de novo assembler from 454 Life Sciences Corporation which is designed to handle their data which is in the form of flowgrams. It is based on the overlap-layout-consensus paradigm and consists of three modules: Overlapper, Unitigger and Multialigner. As 454 doesn't typically produce paired-end data, *Newbler *generates a set of unlinked contigs.

• *EULER *[20] analyzed the feasibility of short read assembly of read length 70–200 using EULER. On simulated data from several bacterial genomes, they produced a mix of long and short contigs.

• EULER-SR [21], the new version of EULER is particularly designed for reads generated by next generation sequencing technologies. The results are based on a hybrid approach where they used 454 and Sanger type data together to generate an assembly. They presented some results for simulated paired 454 reads as well.

• SHRAP [22] is another assembler that assemble reads of length around 200 base pairs using a proposed sequencing protocol for mammalian-scale genomes.

A final class of short-read assemblers focuses on single-ended reads produced by the first generation of Solexa machines:

• SSAKE [1] is a short read assembler that was tested with simulated error-free 25 mers. It performs well with viral genomes. In a recent release, SSAKE started supporting paired end reads.

• SHARCGS [2] is a de novo short read assembler that handles short reads as short as 25–40 bases. It generates a set of large contigs but without any ordering information. Their algorithm was tested against *Illumina's *1 G sequencing instrument. It uses a method that it calls *contig elongation*: a read is extended by looking for other reads in a prefix tree for potential extensions. It doesn't handle paired-end reads.

• *Phusion *[23] was used by *Sanger Institute *to assemble many genomes from shotgun sequences. Recently they showed [24] possibilities of assembling short reads by mixing a low coverage (0.5–2×) of capillary reads with them. They used 454 and Solexa data for their prototype.

• *Adena *[25] is a recently published short read assembler that works on 35 bp long Illumina data and performs better and less resource hungry in comparison to SSAKE and SHARCGS. N50 sizes presented in their paper was in the range 6–14 k bp long.

## Results and Discussion

### Data preparation

We have rigorously tested our assembler on simulated sequence data generated from three bacterial genomes – *M. genitallium*, *S. suis *(strain P1/7) and *E. coli *(DH10B). The reference sequences are 580, 075; 2, 007, 490 and 4, 686, 136 bases long. Our simulations were designed to conform as closely as possible to an assembly project on the Applied Biosystems' SOLiD platform. Indeed our coverage, insert distribution, and base error distribution are derived the actual data set discussed below.

SOLiD is an ultra high-throughput technology. Thus we can exploit the resulting huge coverage to discard bad reads based on the base quality scores [26,27]. After discarding all the reads from the real data set that had average quality scores below 15, we still had 200× of raw reads with a 0.1% base sequencing error rate. The insert length for the data set was found to be normally distributed with mean length of 2,270 and standard deviation 350.

We maintained these properties in our simulated dataset, except that we sampled our reads uniformly whereas the real data set contains artifacts like thinly sampled regions. All reads were 25 bases long and sampled in SOLiD's color space. Starter seeds used in the experiments were around 500 bases long. All data were sampled from both strands of the corresponding genome. All datasets were generated in SOLiD's *two base encoding *format and hence the contigs we generate are also two base encoded.

### Experiments with simulated SOLiD data

Results for one dataset from each of the three species under consideration are shown in Table 2. All the datasets were assembled with 100× input coverage and a small number of starter seeds. The results are shown in different levels of minimum contig accuracy (*S*_*c*_) along with the overall assembly score (*S*_*a*_) which basically shows the quality of our contigs. For a definition of these terms, please see the end of the Methods section.

**Table 2:** Assembly results. Assembly results for different data sets. *Coverage *is the fraction of the reference sequence covered by the generated contigs. Results are shown within different levels of minimum contig accuracy (97%, 99%, 99.9%). Minimum length of a contig considered for this analysis was 100 bases. All contig lengths are expressed in kilo bases. *L*_*c *_is the length of contig *c *whereas *S*_*c *_is its accuracy score. *S*_*a *_is the total assembly score. For further illustration of these terms, refer to the end of the Methods section.

Species	# of reads (million)	# of seeds	Coverage (%)			N50 length (kb)			Max	*S*_*a*_
				
			97	99	99.9	97	99	99.9	*L*_*c *_(*S*_*c*_)	
*M. genitallium*	2.42	5	96.3	88.9	64.6	45.8	36.7	3.5	86.1 (99.1)	96.1

*S. suis*	8.36	7	97.8	93.6	86.3	31.5	25.6	9.5	170.7 (95)	95.7

*E. coli*	19.53	10	98.2	96.1	88.1	41.8	32.4	12	165 (97.4)	97.1

**Figure 1:**
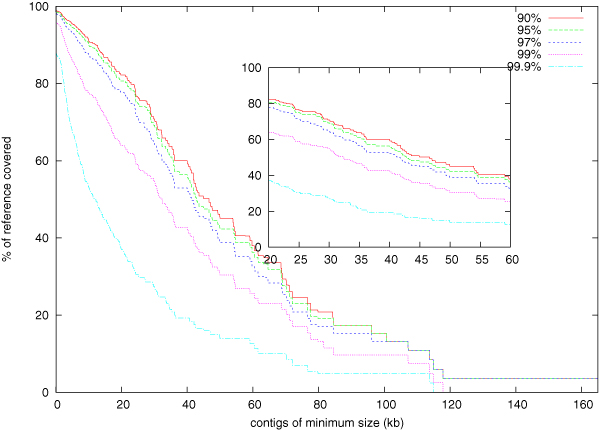
**Min contig len vs. coverage**. Coverage of reference sequence by various sizes of contigs with different levels of minimum contig accuracies (90%, 95%, 97%, 99%, 99.9%; shown in different colors) for the *E. coli *dataset. First portion of the graph (the region containing N50) is zoomed inside the small rectangle.

Table 2 shows the reference coverage achieved along with N50 size generated by SHORTY. In all cases, we achieved over 90% coverage with contig accuracy at least 99%. Our N50 size was also superior than most other comparable assemblers. For *E. coli*, the largest bacteria we considered, we obtained an N50 size of 41.8 kb with contig accuracy at least 97%. The largest contig had a size of 165 kb with an accuracy of 97.4%. Figure 1 shows the coverage achieved by different size of contigs in the *E. coli *dataset. It also shows N90 size with high accuracy (at least 90%) is somewhere around 18 kb.

### Experiments with real SOLiD data

**Figure 2:**
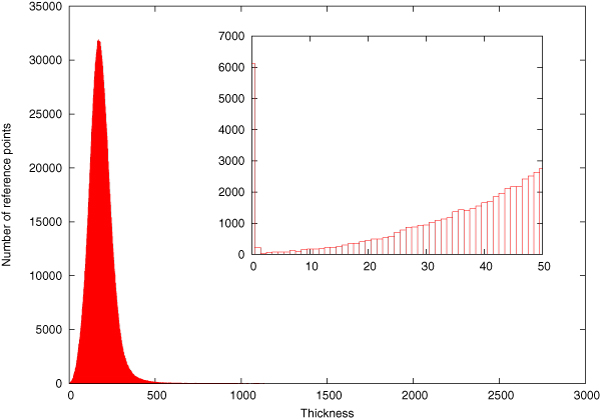
**Thickness in real data**. Frequency of thickness in 200× real SOLiD reads sampled from *E. coli*. Thickness of a position is defined as the number of reads covering that position. The zoomed area shows the areas with low thickness (no more than 50).

We obtained access to an initial real dataset of 300× (read size was 25) coverage of *E. coli *(DH10B) in June 2008. Throwing out the "bad reads" (average base quality score 15 or less) still left us with 200× coverage. After running SHORTY on it, we managed to maintain more than 90% coverage of the reference sequence in pieces of length 100 or more. However the maximum contig length was only 1,641 where the simulated data with half the coverage produced a contig as big as 165,013 bp (Table 2). We ran three experiments (Table 3) with each of them processing three randomly chosen seeds from the reference. All of them show similar results in terms of coverage, contig length and distribution of gaps. Gaps were of short length and uniformly distributed over the sequence. This was due to the thinly sampled regions (see Figure 2) or missing points which we believe is an artifact that needs to be overcome by SOLiD.

**Table 3:** Assembly with real data. Three SHORTY experiments with real ABI SOLiD data. Each experiment was run using three different and randomly sampled seeds. *Coverage *is the fraction of the reference sequence covered by the generated contigs. Results are shown within different levels of minimum contig accuracy (97%, 99%, 99.9%). Minimum length of a contig considered for this analysis was 100 bases. For gap analysis, minimum contigs accuracy considered was 90%. *L*_*c *_is the length of contig *c *whereas *S*_*a *_is the total assembly score. For further illustration of these terms, refer to the end of Section "Methods".

Test	Coverage (%)			Average *L*_*c*_	# of gaps	Avg. gap len	*S*_*a*_
					
	97	99	99.9				
1	92.3	87.8	57.4	161.5	4691	62.3	94.4

2	93.3	88.4	58.7	164.5	4736	58.5	94.5

3	92.9	88.1	58.2	165.0	4690	63.4	94.5

To put our performance on the real dataset into perspective, we tested the data against other available assemblers. None of them currently works with ABI's *color space *data, making it difficult to find suitable assembler that could run SOLiD data with minor format change. We tried SHARCGS [2], but even the 32 G memory available in our machines did not suffice for assembly. We tried the latest version of SSAKE [1] with the default setting for paired end reads. SSAKE produced only 846 contigs (only 2.1% coverage of the reference sequence) that were longer than 100 bases, with the maximum contig size of 337 bp.

### Resource consumption

All experiments mentioned here were running in machines with two 1.8 GHz AMD Opteron processors. In the end, SHORTY was proved to be extremely efficient in memory consumption. A 100× single seeded experiment with *M. genitallium *took 220 M of memory. Experiments with *S. suis *and *E. coli *consumed 1.2 G and 2.7 G memory respectively. Their corresponding running time was 3, 6 and 10 hours. When using multiple seeds, we run each seed in parallel in different machines. That means, SHORTY is capable of running in most of today's personal computers. We have seen that resource consumption was one of the major concerns with short read assembly due the the huge volume of data.

## Conclusion

We have presented what we believe to be the first results on the de novo assembly of ABI SOLiD data. The results we have presented here provide evidence that high-quality short read assembly is indeed possible using simple and economical protocols on real short-read data. Unlike previous works, our protocol uses a single sample preparation as opposed to a mix of insert sizes or or runs on a mix of different platforms (e.g. 454 and Solexa). Our assemblies thrive on significant variance in insert length, further simplifying preparation over others in the literature.

Our use of single seed reads is more of a nuisance than a problem, as this data can be obtained cheaply through outsourcing services. An interesting question is whether they are really necessary. Database sequence from closely-related species should suffice, but even more to the point is noting how little information they add to the process. Three 500 base seeds represent only 3,000 bits of information in an assembled genome of 4,000,000 bits, making it hard to believe they really are essential. Our proposed alternative to this is to run an existing assembler on the initial reads to get few contigs large enough to serve as starter seeds. We hope add this to SHORTY as part of a future release.

Our primary direction of further work is demonstrating significant de novo assemblies on each of the major short-read platforms, namely ABI SOLiD™, Solexa paired-read data, and Helicos Biosciences data as they become available to us. We are also working to raise our N50 sizes through gap filling techniques based on accurate positional estimation.

## Methods

SHORTY is designed to work on paired end short reads. These reads (optionally accompanied by quality scores) are generated by recent technologies developed by Applied Biosystems, Solexa and others to come. Laboratory protocols aim to select targets whose insert separating the two reads is normally distributed around a given target length. Each paired read contains a distinct *left-mer *(*lmer*) and a *right-mer *(*rmer*). These reads may contain insertion, deletion, substitution or homo-polymer errors. Our protocol also uses a few 300 – 500 bp long reads (we call them *seeds*) to start with. Here is the basic workflow of SHORTY algorithm which is followed by a detailed description of each step:

1. Reads are stored in an efficient data structure to be able to be retrieved later. Also all bad reads are discarded.

2. A *seed *is chosen and processed. This can be any of the *starter-seeds *or *next generation *seeds that SHORTY generates. This step basically gives us a set of neighboring reads whose other ends (from the mate-pair) map onto the seed.

3. From the group of reads we got from the previous step, we generate contigs based on their overlap information. We discard contigs that are below certain threshold length (say 100 bases).

4. Some of the contigs generated are long enough to be considered as seeds again. For each such contig, we repeat from step 2. If we don't have any such contigs, we continue to the next step.

5. All contigs generated from all seeds (including multiple starter seeds) are considered together for further processing to generate larger contigs.

6. Scaffolds are generated and inter-contig gaps are calculated. Also, mis-assemblies (e.g. chimera) are detected at this step.

### Read storage

In a typical data set, we have millions of pairs of reads. Our use of these reads requires them to be accessed many times during the assembly process. To make the searching process faster while spending less memory, we store the reads in a compact trie. We insert all the pairs based on their *left-mers *and *right-mers*. From each pair, we generate another pair of reads. The *left-mer *of the new pair will be the reverse complement of the *left-mer *of the original pair. Similarly the *right-mer *of the new pair will be the reverse complement of the *right-mer *of the original pair. We insert the new pair in a similar way. So, after the trie building process is finished, we know for a particular *k-mer *which pairs of reads contain that *k-mer *in their *left-mer *or *right-mer*.

Since reads contain a variety of sequencing errors, our trie searching method is designed to search a *k-mer *within a certain number of maximum mismatches allowed.

### Processing a seed

**Figure 3:**
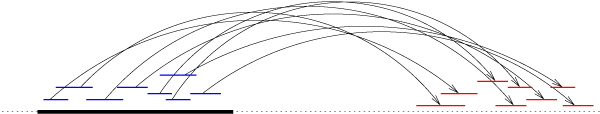
**Seed processing**. Group of *right-mers *(red) whose corresponding *left-mers *(blue) can be mapped on the *seed *(black). It can also be in the other way, i.e. *right-mers *mapped on the *seed *to form a group of *left-mers*.

**Figure 4:**
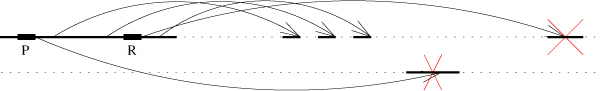
**Repeats and palindromes**. Repeats (R) and palindromes (P) can generate contigs with misleading positional information.

For each *seed *we detect the group of read pairs whose *left-mers *(or *right-mers*) will map onto that seed (by searching the trie we have already built). With high probability, this group of *right-mers *(or *left-mers*) belong to some neighboring reads in the reference sequence (Figure 3). The previously built trie of reads is used to determine this group. We take into consideration various types of sequencing errors while trying to map a read on the *seed*. This group forms the basis of forming larger contigs and next generation *seeds*. While processing a seed, we carefully try to handle repeat regions (Figure 4) as these might generate contigs with misleading positional information. Another situation where this problem can arise is when there are regions which are similar to their reverse complement. We call such regions *palindrome *regions. We detect seeds with repeats or palindrome regions by checking the number of reads that a seed can map onto itself. If it attracts too many reads (say twice as much as the input coverage), there is a strong chance that it has one of those problems.

### Generating contigs

The group of reads formed in the previous stage is expected to have overlapping reads when we are presented with enough input coverage. We construct a directed graph *G *= (*V*, *E*) where *V *is the set of reads in the group. *E *contains all the edges in the graph. An edge *e*_*ij *_∈ *E *if there is a qualified (say above some threshold) overlap from read *i *to *j*. *e*_*ij *_denotes the amount of overlap (For 25 base long SOLiD reads, we considered an overlap if it were at least 15 base long. But it depends on the coverage and quality of the data). We greedily choose the longest path from *G *and merge the reads along that path to produce a contig. We keep doing this for the next longest path possible. We only consider paths longer than a threshold. This generates a set of contigs which are expected to be in neighboring positions in the reference. While considering an overlap, we also take into account the quality of the bases. Considering the possibility that the reads may contain different kinds of errors, it is not always possible to have 'clean' overlaps. We use a *dynamic programming *based approach where we penalize for such error conditions. We also store voting information (for each position we count the number of A, T, G and C that comes from the constituting reads) for each position of a contig which helps us to choose the appropriate base for a position in case we have multiple candidates which might be the result of errors in the constituting reads. This can be used to determine the quality of that base.

### Generating new seeds

Some of the *contigs *generated in the previous stage might qualify to be used as *seeds *again. These *next generation seeds *are chosen based on the length and quality of the *contigs*. It is also possible to use a collection of neighboring *contigs *as a single *seed*. However, in SHORTY we only consider individual *contigs *as *seeds*.

New seeds are treated like starter seeds. This is where SHORTY keeps iterating until we have no more seeds or reads available for use or we have reached our desired output thickness level.

### Generating larger contigs

At this stage we are expected to have decent sized *contigs*. In SHORTY, we allow a single read to be used multiple times to generate redundancy of contigs. This compensates for sequence repetitions, and works well when the data is sampled uniformly with few gaps, as should happen when sequencing with high coverage. We define output thickness defined as the ratio of the total length of generated contigs and reference sequence length. If our target output thickness is *t*, we usually allow a read to be used maximum *t *times. A typical value of *t *for a single starter seeded run is 5. With more starter seeds, we can choose a lower value of *t*. With *t *> 1, it is expected that contigs will overlap among themselves. This along with the normal distribution of insert lengths form the basis of forming larger contigs and removal of inter-contig gaps.

**Figure 5:**
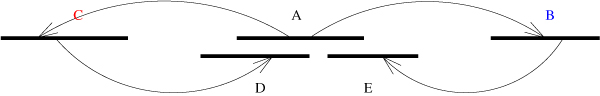
**Narrowing gaps**. Because of the standard deviation of insert-length, gap between successive *contigs *reduces.

Figure 5 explains how the gap between *contigs *get reduced. Contigs *B *and *C *are generated from seed *A*. If the standard deviation of *insert length *was zero, *D*, which is generated from *C*, must have overlapped completely with *A*. But we can safely assume that we will have 10–15% standard deviation in actual situation. However, we can still proceed in a situation where the standard deviation is zero using multiple *seeds*. As we can see in Figure 5*A*, *D *and *E *now overlap to produce a larger *contig *while also reducing the gaps.

The number of contigs available for processing in this stage is very large. For example, a SHORTY run of *E. coli *with desired output thickness 5 and read reuse limit 5 produces more than 90, 000 contigs. Finding candidate merges like Figure 5 from this large amount of possibilities can be expensive. Also, with the presence of repeat regions, considering a merge based on overlaps without any other information might be misleading.

**Figure 6:**
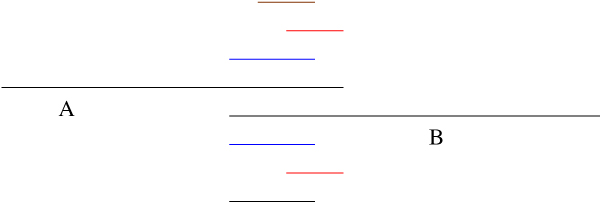
**Contig overlap**. Overlap between two contigs is only considered when reads with similar ids from both contigs make this. Here blue and red reads appear in both contigs and can form this overlap.

But SHORTY keeps track of reads that made the contigs. Also, because of the way we generate a contig, with very high probability constituting reads are not fooled by repeats and are from the neighboring locations. We use this information to merge these contigs. At first we construct a directed graph *G *= (*V*, *E*) like before (see Section "seed processing"). But in this graph *V *contains all the contigs and *E *contains all the edges in the graph. Again, an edge *e*_*ij *_represents an overlap from contig *i *to contig *j*. But unlike the overlap calculation used in "seed processing", here overlaps don't represent a string overlap only. An overlap here also indicates that a common set of reads (Figure 6) made the overlapping portion in both contigs. This way we are able to avoid many repeat related issues. Once we have the graph, we keep extracting the longest possible paths from *G *using the previously used greedy algorithm. This also increased our contigs' size significantly.

### Contig scaffolding

**Figure 7:**
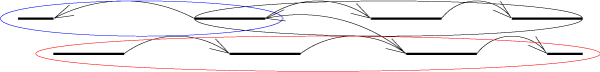
**Scaffold construction**. A chain of contigs in the same direction is used as a scaffold. Three different scaffold are shown in this figure in red, blue and black.

We use mate pair data in such a way that it is possible to trace back which contig was generated from which seed (Figure 7). We construct a directed graph *G *= (*V*, *E*) from this information. Vertices of *G *are the contigs and each seed is connected to all of its children. An edge *e*_*ij *_is a directed edge from *i *to *j *if *i *is the generating seed for *j*. A chain of contigs in the same direction in the graph forms a scaffold.

**Figure 8:**
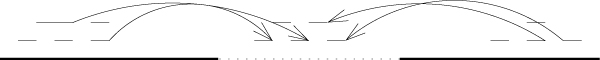
**Scaffold gap removal**. Unfilled gaps in scaffolds can be filled by re-mapping the reads onto the contigs.

Contiguous contigs in the scaffold are merged if they become large enough in the previous steps. We can also re-map our initial reads to fill the gaps in a scaffold (Figure 8). If the gaps still persist, we provide an estimated gap length (see "Estimating gap between contigs").

### Detecting misassemblies

**Figure 9:**
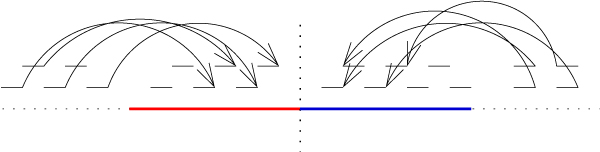
**Chimera detection from read pairs**. *Chimeras *are detected by mapping back the reads onto the contig. The contig is partitioned at a point where only a few pair have their reads in both sides of that point.

**Figure 10:**
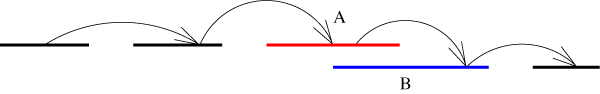
**Chimera detection from scaffold**. Contigs shown here are from one scaffold. *A *and *B *are two consecutive contigs large enough to overlap. If they don't overlap, there is a chance that either *A *or *B *is a chimera produced due to mis-assembly.

We detect mis-assemblies by mapping the reads back to the contigs (Figure 9). If there is a point where a wrong merge occurred, there are only a few paired reads where both of them get mapped to the opposite sides of that point. We split the contigs in such points. Our scaffolds can also be another source of identifying *chimeras *(Figure 10). Our scaffolds are ordered chains of contigs generated from mate paired reads. When two contigs become large enough to cover the gap between them, they should ovelap. Otherwise, there is a chance that at least one of them is a *chimera*.

### Estimating gap between contigs

The combination of relatively high-coverage as realized by paired-end microreads provides new opportunities to accurately estimate the distance between non-overlapping contigs (e.g. Figure 8). The simulations discussed in this paper assume 100× sequence coverage in 25-base paired reads. This yields an expectation of two reads starting from each position on the genome, half of which will represent the 5' read. This implies that the number of read-pairs spanning any interior position on the genome roughly equals the insert length. Thus hundreds or even thousands of read pairs connect each two non-overlapping contigs, all of whose insert sizes were drawn from a normal distribution of known mean and standard deviation. By analyzing where these read-pairs map on each contig we can accurately estimate the inter-contig distance. Huson et. al [3] has the statistical analysis required to calculate such distances and we use similar calculations for SHORTY.

Accurate distance estimation is vital in later-stage contig merging in SHORTY. Many contigs generated from seeds overlap, but too weakly to be statistically significant over the scale of a genome assembly. Accurate information about position enables us to merge them confidently. Secondary benefits include reduced running times (by avoiding unpromising contig-overlap pairs) and dealing with repeats.

### Contig accuracy

To verify how well SHORTY is performing we determine the accuracy of a contig by mapping it back to the reference genome. MUMmer [28], which uses an efficient method based on suffix tree, is used to find these matches. For a match, an accuracy score *S*_*c *_is defined as *x *+ *y *- 100, where *x*% of contig *c *was matched to the reference sequence with *y*% similarity. This way, we highly penalize for mismatched parts of a contig and only very good contigs get a good score. This is in contrast to some other assemblers where accuracy is only based on similarity and low penalty for mismatches.

### Assembly score

SHORTY produces some misassembled contigs, just like any other assembler. In order to determine the overall assembly quality, these bad contigs should be taken into account. Unlike most other methods (where % of good contigs are reported) used in the literature, we take an weighted scoring scheme where alonger contig has more influence than a shorter contig. The total assembly score *S*_*a *_is defined as (∑_∀*c*∈*C *_*S*_*c*_*L*_*c*_)/∑_∀*c*∈*C *_*L*_*c*_, where *C *is the set of all contigs, *S*_*c *_is the accuracy measure for contig *c *∈ *C *and *L*_*c *_is the length of contig *c *∈ *C*.

## Availability

All documentation, source code and other information on SHORTY are available at .

## Competing interests

The authors declare that they have no competing interests.

## Authors' contributions

MSH was responsible for the overall design and implementation of SHORTY and drafted the manuscript. NA made significant contributions in designing certain key data structures and contig geography. SS was involved in the overall design and drafting the manuscript. All authors reviewed and approved the final manuscript.
